# Regularization Techniques for ECG Imaging during Atrial Fibrillation: A Computational Study

**DOI:** 10.3389/fphys.2016.00466

**Published:** 2016-10-14

**Authors:** Carlos Figuera, Víctor Suárez-Gutiérrez, Ismael Hernández-Romero, Miguel Rodrigo, Alejandro Liberos, Felipe Atienza, María S. Guillem, Óscar Barquero-Pérez, Andreu M. Climent, Felipe Alonso-Atienza

**Affiliations:** ^1^Department of Telecommunication Engineering, Universidad Rey Juan CarlosFuenlabrada, Spain; ^2^ITACA, Universitat Politécnica de ValenciaValencia, Spain; ^3^Instituto de Investigación Sanitaria Gregorio Marañón, Hospital General Univesitario Gregorio Marañón, Universidad Complutense-Facultad de MedicinaMadrid, Spain

**Keywords:** atrial fibrillation, ECG imaging, regularization, dominant frequency, rotor location, inverse problem

## Abstract

The inverse problem of electrocardiography is usually analyzed during stationary rhythms. However, the performance of the regularization methods under fibrillatory conditions has not been fully studied. In this work, we assessed different regularization techniques during atrial fibrillation (AF) for estimating four target parameters, namely, epicardial potentials, dominant frequency (DF), phase maps, and singularity point (SP) location. We use a realistic mathematical model of atria and torso anatomy with three different electrical activity patterns (i.e., sinus rhythm, simple AF, and complex AF). Body surface potentials (BSP) were simulated using Boundary Element Method and corrupted with white Gaussian noise of different powers. Noisy BSPs were used to obtain the epicardial potentials on the atrial surface, using 14 different regularization techniques. DF, phase maps, and SP location were computed from estimated epicardial potentials. Inverse solutions were evaluated using a set of performance metrics adapted to each clinical target. For the case of SP location, an assessment methodology based on the spatial mass function of the SP location, and four spatial error metrics was proposed. The role of the regularization parameter for Tikhonov-based methods, and the effect of noise level and imperfections in the knowledge of the transfer matrix were also addressed. Results showed that the Bayes maximum-a-posteriori method clearly outperforms the rest of the techniques but requires a priori information about the epicardial potentials. Among the purely non-invasive techniques, Tikhonov-based methods performed as well as more complex techniques in realistic fibrillatory conditions, with a slight gain between 0.02 and 0.2 in terms of the correlation coefficient. Also, the use of a constant regularization parameter may be advisable since the performance was similar to that obtained with a variable parameter (indeed there was no difference for the zero-order Tikhonov method in complex fibrillatory conditions). Regarding the different targets, DF and SP location estimation were more robust with respect to pattern complexity and noise, and most algorithms provided a reasonable estimation of these parameters, even when the epicardial potentials estimation was inaccurate. Finally, the proposed evaluation procedure and metrics represent a suitable framework for techniques benchmarking and provide useful insights for the clinical practice.

## 1. Introduction

Atrial fibrillation (AF) is the most common arrhythmia in clinical practice affecting up to 33 million patients (Burstein and Nattel, 2008) and is associated with an increased risk of embolism, cardiac failure, and mortality (Fuster et al., 2006). Ablation strategies for AF are based on the electrical isolation of atrial tissue responsible for the initiation or maintenance of the fibrillatory process (Guillem et al., 2016). Recently, some works have applied the inverse problem of electrocardiography or electrocardiographic imaging (ECGI) to reconstruct epicardial potentials during AF and even guide AF ablation procedures (Cuculich et al., 2010; Pedrón-Torrecilla et al., 2016).

The ECGI aims to non-invasively reconstruct the electrophysiological activity on the heart surface from BSP (Brooks and Macleod, 1997; Gulrajani, 1998). Methodologically, the ECGI combines signal-processing approaches with numerical modeling of the bioelectric properties of the patient's thorax, and is formally formulated as an inverse problem (Rudy and Messinger-Rapport, 1988). Even if the propagation mechanism between the epicardium and the torso is perfectly modeled, the ECGI poses a complex problem that is generally ill-posed, since a lot of information is lost during the propagation of the signal (Rodrigo et al., 2014). Therefore, regularization methods have been applied with promising results in order to obtain stable and realistic solutions (Tikhonov and Arsenin, 1977; Oster and Rudy, 1997; Pedrón-Torrecilla et al., 2011; Shah et al., 2013). Thus, the ECGI may be used to recover cardiac epicardial potentials (MacLeod and Brooks, 1998; Oosterom, 2012; Milanič et al., 2014), activation sequences (van Dam et al., 2009), arrhythmogenic substrates (Cuculich et al., 2011; Álvarez et al., 2012; Rudy, 2013; Wang et al., 2013), or dominant high-frequency regions (Pedrón-Torrecilla et al., 2016). The ECGI technology is becoming clinically relevant for analyzing irregular propagation patterns, such as during AF. In this setup, phase mapping techniques have been used to analyze the spatio-temporal structure of the signal (Gray et al., 1998; Zlochiver et al., 2008; Rodrigo et al., 2014). However, the ECGI has not been fully validated during these non-stationary propagation patterns. In fact, the clinical application of ECGI during AF results in simple activation patterns that do not correspond to the expected complex propagation patterns recorded in patients (Cuculich et al., 2010). One of the reasons for this lack of systematic validation of ECGI during AF is the lack of standard target parameters for comparison. Most of the works related to the ECGI focus on comparing the spatial or temporal pattern of the electrical activity in terms of a given similarity metric, like relative error, correlation coefficient (CC), or root mean square error (RMSE) (Serinagaoglu et al., 2006; Milanič et al., 2014). More recently several works compared the spectral properties of the electrical activity in terms of the dominant frequency (DF) (Pedrón-Torrecilla et al., 2016). In summary, independent studies have proposed and compared algorithms for estimating different target parameters (e.g., surface potentials, activation times, DF, SP location), and have used diverse performance metrics that have not always a clear application in the clinical practice.

Therefore, the ability of ECGI to accurately retrieve complex epicardial propagation patterns from the patterns on the torso, and the procedure to assess the quality of the results, need to be further explored. This study presents a systematic assessment of different regularization techniques during AF, aiming to: (i) compare their performance for several target parameters; (ii) identify reliable and easy-to-interpret performance metrics for each target parameter; and (iii) analyze the effect in performance of different parameters, like the complexity of the propagation pattern, the SNR and the accuracy of the regularization parameter computation. Furthermore, this study aims at providing a unified framework for inverse methods benchmarking by making available all the routines and data at http://www.tsc.urjc.es/~carlos.figuera/ECGI_in_AF/.

The rest of the paper is organized as follows. The second section presents the methods used for the computational experiments, including the models, regularization techniques, the target parameters, the performance metrics, and the experimental setup. Results are described in Section 3 and discussed in Section 4. Finally, Section 5 summarizes the main conclusions of this work.

## 2. Methods

### 2.1. Computerized models and forward problem

**Figure 1:**
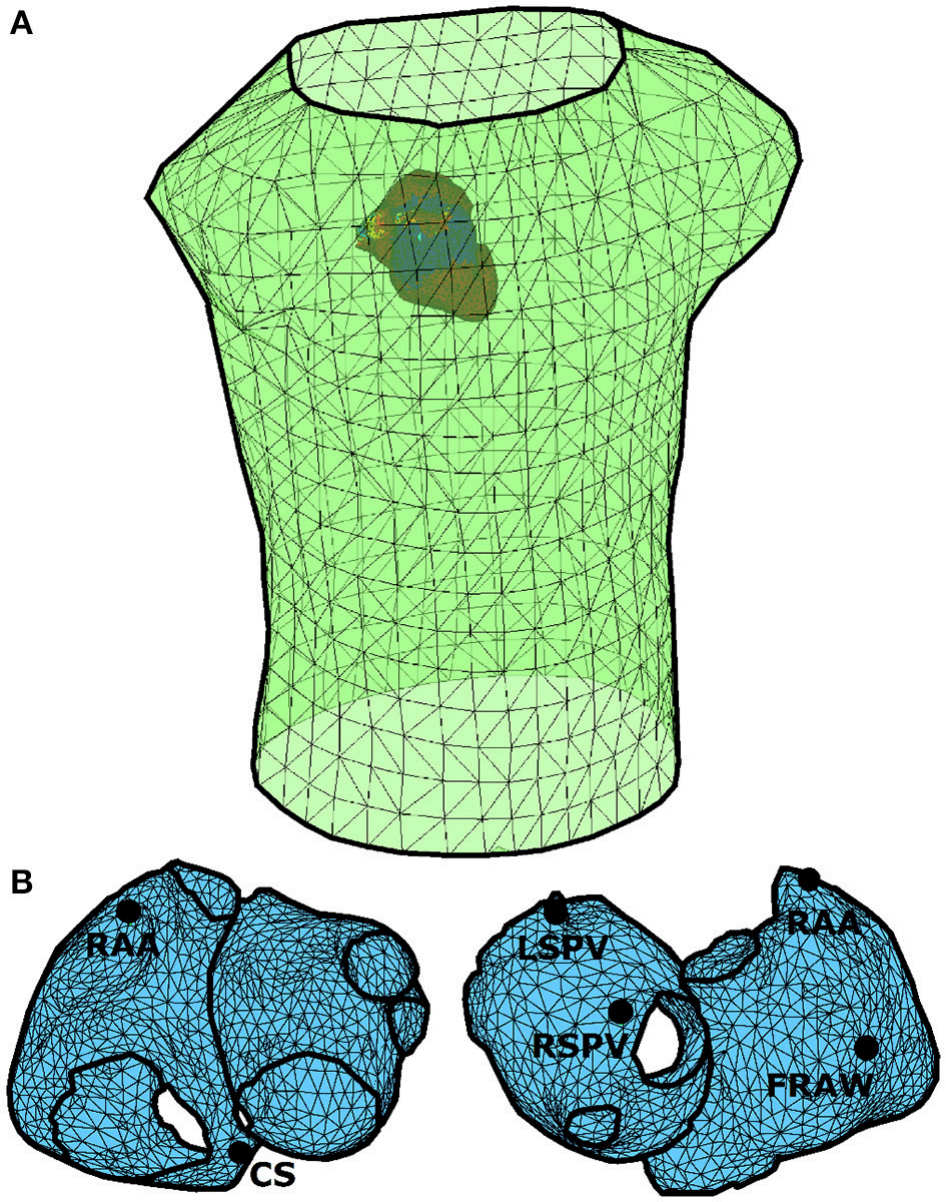
**Anatomical models of the atria (***N*** = 2039 nodes) and torso (***M*** = 659 nodes)**. **(A)** Spatial configuration of the torso and atria models. **(B)** Anatomical model of atria in which five clinically relevant points have been selected: right atrial appendage (RAA), coronary sinus (CS), left superior pulmonary vein (LSPV), right superior pulmonary vein (RSPV), and free right atrial wall (FRAW).

Forward problem is simulated by using realistic computerized models of atria and torso (Pedrón-Torrecilla et al., 2016). Figure 1 shows the geometry of these models with *N* = 2039 nodes in the atria and *M* = 659 nodes in the torso, and highlights five clinically relevant points on the atria that are used in this work: right atrial appendage (RAA), coronary sinus (CS), left superior pulmonary vein (LSPV), right superior pulmonary vein (RSPV), and free right atrial wall (FRAW). A single torso model is used in order to reduce potential sources of noise during the comparison of methods. Since, as described in Zemzemi et al. (2015), noise level in clinical practice hides the effect of torso heterogeneity on the inverse solution, this does not imply a loss of generality of the simulation results. These models allow us to simulate different propagation patterns over the atrial surface and the associated BSP (García Mollá et al., 2014), from which the epicardial potentials are inversely calculated using different regularization methods.

**Figure 2:**
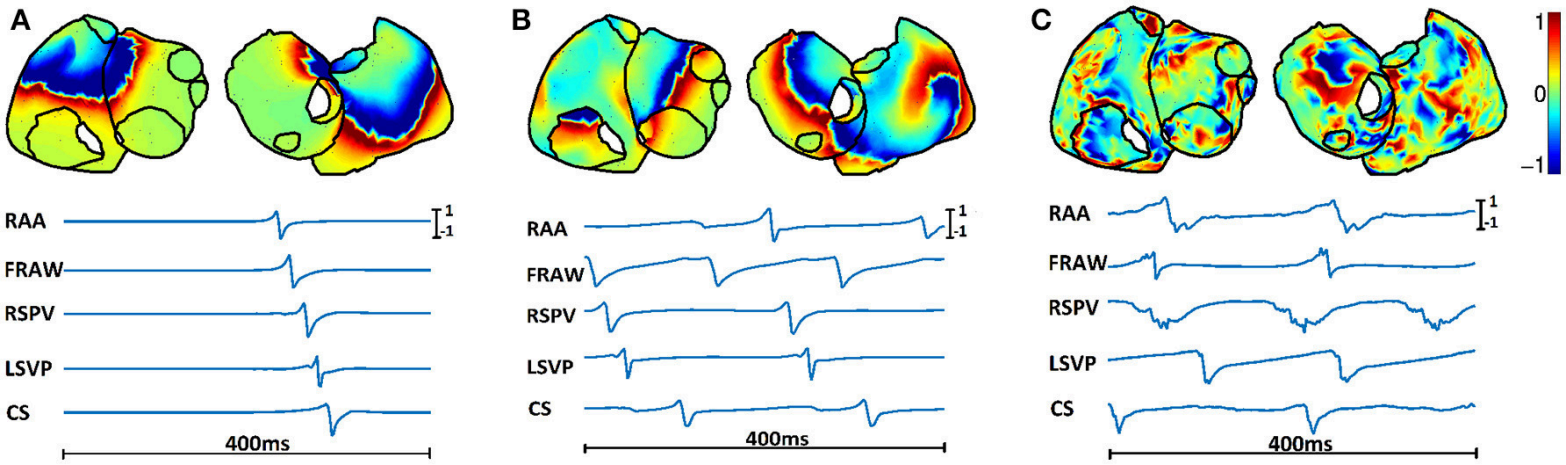
**Spatial distribution (top) and temporal evolution (bottom) of epicardial potentials for three propagation patterns: (A) SR, (B) SAF, and (C) CAF**. Time signals are shown for the five points highlighted in Figure 1.

Three propagation patterns are considered:

- Normal sinus rhythm (SR, see Figure 2A), in which the atrial tissue is periodically activated at 1.2*Hz*.
- Simple AF propagation pattern (SAF), represented by a right-to-left DF gradient (Figure 2B). This scenario is simulated by a single functional reentry located in the right atria, which rotates at 7.3*Hz*. The rest of the atrial tissue (left atria) is activated at 4.7*Hz*.
- Complex AF propagation pattern (CAF), with 25% of atrial cells being under fibrotic conditions (Figure 2C) (Rodrigo et al., 2016). A single functional reentry is simulated near the RSPV. In the surrounding area of the rotor, there is rotational electrical activity at 6.8*Hz* while the rest of the atria activates at 5.4*Hz*.

**Figure 3:**
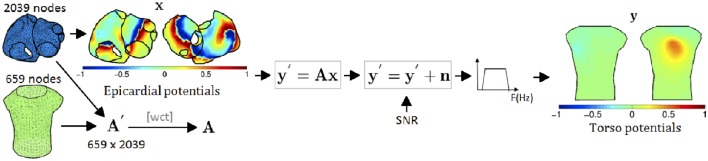
**Process for the forward problem: (1) torso potentials are computed using BEM; (2) transfer matrix is transformed to account for the WCT reference used for the BSPs; (3) noise is added; and (4) torso signal is filtered**.

Signal propagation between the atria and torso is simulated by using the boundary element method in order to obtain the *M* × *N* transfer matrix *A*′ (Barr et al., 1977; de Munck, 1992; Horácek et al., 1997; Stenroos and Haueisen, 2008; Pedrón-Torrecilla et al., 2016). Once *A*′ is known, the BSPs are computed as shown in Figure 3. First, the BSPs are computed for each time instant as $y_{t}^{\prime }=Ax_{t}$, where $y_{t}^{\prime }$ and *x*_*t*_ represent the BSPs and the epicardial potentials at time *t*, respectively, and *A* is a transformation of *A*′ that accounts for the use of Wilson Center Terminal (WCT) for referencing the BSPs. Then, $y_{t}^{\prime }$ is corrupted with additive Gaussian noise and filtered using a fourth order Butterworth filter with cutoff frequencies 3 and 30 Hz for fibrillatory models and 0 and 30 Hz for SR, obtaining the BSPs vector *y*_*t*_.

### 2.2. Inverse problem

**Figure 4:**
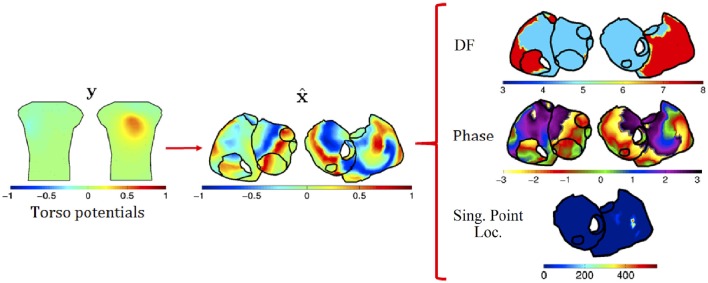
**Process for the inverse problem: (1) regularization techniques are applied to BSPs to obtain epicardial potentials; and (2) from these, DF, phase, and SP location are computed**.

The whole process is summarized in Figure 4. First, the epicardial potentials are estimated using a regularization method. Second, the DF, phase maps, and SP location are computed from the estimated epicardial potentials. For the first step, we assume the following linear model

$$y_{t}=Ax_{t}+\epsilon ,$$

where **ϵ** represents the model residuals. The target is to estimate the epicardial potentials *x*_*t*_ at time instant *t*, from measurements at the torso *y*_*t*_ with the knowledge of *A*. This problem is ill-conditioned and hence a plethora of regularization methods have been proposed to solve it. We next summarize the ones that are considered in this work.

#### 2.2.1. Tikhonov regularization

Tikhonov regularization is usually employed in linear inverse problems to stabilize the solution by penalizing its complexity. For obtaining the epicardial potentials *x*_*t*_ at instant *t*, the functional to minimize is:

(1) $$\begin{array}{l} ||y_{t}-Ax_{t}|{|}_{2}^{2}+\lambda_{t}||Lx_{t}|{|}_{2}^{2} \end{array}$$

where *y*_*t*_ is the vector containing the torso measurements at instant *t*, λ_*t*_ is the regularization parameter for that time instant, and *L* is a *N* × *N* matrix that can take three forms: identity matrix (zero-order Tikhonov, which minimizes the *L*_2_-norm of the solution); Gradient operator (first-order Tikhonov, which favors flat, i.e., constant, solutions, and penalizes gradients); and Laplacian operator (second-order Tikhonov, which favors smooth, i.e., constant-gradient, solutions). The solution of this problem is:

(2) $$\begin{array}{l} {\hat{x}}_{t}={\left(A^{T}A+\lambda_{t}\;L^{T}L\right)}^{-1}A^{T}y_{t} \end{array}$$

By stacking all the measurements in a *M* × *T* matrix *Y*, with *T* the number of time instants, the problem can be solved at once by minimizing:

(3) $$\begin{array}{l} ||Y-AX|{|}_{Fro}^{2}+\lambda_{g}||LX|{|}_{2}^{2} \end{array}$$

where *X* is a *N* × *T* matrix with columns *x*_*t*_, λ_*g*_ is a constant global regularization parameter that does not depend on time, and ||·||_*Fro*_ stands for the Frobenius norm. The solution is similar to Equation (2) but substituting ${\hat{x}}_{t}$, *y*_*t*_, and λ_*t*_ with $\hat{X}$, *Y*, and λ_*g*_, respectively.

#### 2.2.2. Truncated and damped singular value decomposition (TSVD and DSVD)

The solution to zero-order Tikhonov method can be formulated in terms of the SVD of the transfer matrix, *A*. The regularization acts a filter of the singular values σ_*r*_ (with *r* = 1…*R*, being *R* the rank of *A*) of the transfer matrix that attenuates the smallest ones. The filtering function for each σ_*r*_ is $\frac{\sigma_{r}^{2}}{\sigma_{r}^{2}+\lambda_{t}}$ (Hansen, 2007).

TSVD and DSVD also regularize the problem by filtering the singular values of *A*. The DSVD regularization filters the smallest singular values more smoothly than the Tikhonov method, i.e., the filtering function is $\frac{\sigma_{l}}{\sigma_{l}+\lambda_{DSVD}}$. The free parameter λ_*DSVD*_ is a free parameter that must be set a priori. With TSVD the smallest singular values of the decomposition of the transfer matrix *A* are ignored (Hansen, 1998, 2007), and then the execution time is shortened. The number of discarded singular values is a free parameter that must be set a priori. The modified TSVD algorithm (Hansen et al., 1992) allows to extend the SVD-based regularization to the first and second order Tikhonov (i.e., with *L* being the Gradient and Laplacian operators, respectively). To explicitly reference the order, in this work the three TSVD methods are named TSVD-0, TSVD-1, and TSVD-2.

#### 2.2.3. Total variation (TV)

Instead of the *L*_2_-norm regularization applied by the Tikhonov-based methods, a *L*_1_-norm penalization of the gradient function was applied in Ghosh and Rudy (2009). The objective was to favor more detailed and less-smoothed solutions. The function to minimize is

(4) $$\begin{array}{l} ||y_{t}-Ax_{t}|{|}_{2}+\lambda_{t}||Lx_{t}|{|}_{1} \end{array}$$

where ||·||_1_ is the *L*_1_-norm, and *L* is the Gradient matrix.

#### 2.2.4. Bayesian maximum a posteriori estimation (Bayes)

If the spatial covariance matrix and the mean of the epicardial potentials are known, they can be included as a priori information by making use of the Bayesian MAP estimator (van Oosterom, 1999; Serinagaoglu et al., 2005). Assuming zero mean for the epicardial potentials, the solution is:

(5) $$\begin{array}{l} {\hat{x}}_{t}=\left(C_{x}A^{T}\right){\left(AC_{x}A^{T}+C_{n}\right)}^{-1}y_{t} \end{array}$$

where ***C***_*x*_ and ***C***_*n*_ are the covariance matrices of the epicardial potentials and noise, respectively. In order to estimate ***C***_*x*_ without committing inverse crime several approaches can be followed. In Serinagaoglu et al. (2002; 2006) the authors use sparse measurements of the epicardial potentials made with multielectrode coronary venous catheters, and in Hanna et al. (2009) many forward problem results are used as a priori information for the maximum a posteriori estimator.

#### 2.2.5. Greensite (GS)

The MAP approach only accounts for spatial correlation of the potentials. The temporal correlation can also be included in the problem by using the isotropy assumption (Greensite, 2003). Then, the spatio-temporal covariance matrix can be computed as ***C***_*X*_ = ***C***_*t*_ ⊗ ***C***_*x*_, where ***C***_*x*_ is the spatial covariance matrix and *C*_*t*_ is the temporal covariance matrix. Since ***C***_*X*_ is large, a whitening filter can be applied to the data, and the problem is then solved instant by instant with the MAP approach (Onal and Serinagaoglu, 2009). We use the method in Onal and Serinagaoglu (2009), where the whitening filter is combined with a Tikhonov approach.

#### 2.2.6. Generalized minimal residual (GMRES)

The additional penalizations in the functionals of Tikhonov-based methods and TV, and the MAP-based formulations rely on having a certain amount of a priori information about the solution. GMRES method aims to avoid any additional constraints or assumption about the solution of the inverse problem (Ramanathan et al., 2003). Since it is an iterative method, the regularization is obtained by limiting the number of iterations (Saad and Schultz, 1986).

### 2.3. Target parameters

Usually, the objective of the inverse problem computation is to estimate the epicardial potentials. However, during AF, several more targets can be considered, and might be indeed clinically more relevant than the raw potentials. We now present the whole set of target parameters that have been estimated, along with a brief description of the methodology used to compute them.

#### 2.3.1. Epicardial potentials

The computational models used in this work depart from monopolar epicardial potentials as the origin of the electrical activity. This spatio-temporal signal is directly estimated by applying the inverse methods described in Section 2.2.

#### 2.3.2. Dominant frequency

DF has become a very useful tool in the clinical practice since it can be used as a target for the ablation procedure (Guillem et al., 2013; Atienza et al., 2014). Then, once epicardial potentials are estimated, frequency analysis is performed: a Welch periodogram (2 s Hamming window, 50% overlap, sampling rate 500 Hz) is used to estimate the power spectral density at each node. Then, harmonics are discarded and the resultant highest peak is selected (Guillem et al., 2013).

#### 2.3.3. Phases

Signals are filtered with a band-pass filter around the DF (passband from 3 Hz to DF + 2 Hz). A Hilbert transform is then applied to compute the phase in each node (Rodrigo et al., 2014).

#### 2.3.4. Singularity point (SP) location

Phase maps are used to detect the core of the reentrant activity (Rodrigo et al., 2014; Guillem et al., 2016). Ablation of SPs has been proposed as a novel technique for the termination of AF, both from invasive (Narayan et al., 2012, 2013) and non-invasive (Shah et al., 2013) recordings. A SP is defined as the point in a phase map that is surrounded by phases from 0 to 2π. Only those SPs that are present for the duration of at least two full rotations are considered (Rodrigo et al., 2016). With this method, none, one or more singular points are estimated for each time instant. Finally, dominant SP is defined as the one located in the highest DF area.

### 2.4. Performance metrics

Now we describe the performance metrics used for benchmarking the inverse methods for the different target parameters. The proposed metrics focus either on temporal aspects of the signals (epicardial potential estimate) or spectral (and related) aspects of the signals (DF, phase maps, and SP location). To quantify the performance metrics related to each of the clinical targets, we use as gold standard the actual epicardial potentials filtered with the same filter as the torso potentials. For the rest of the target parameters (frequency, phase, and SP location) the gold standard is obtained by applying the procedures described in Sections 2.4.2 and 2.4.3 to the actual epicardial potential.

#### 2.4.1. Epicardial potential metrics

To measure the similarity between the real (***x***) and estimated ($\hat{x}$) epicardial potentials, we use the Pearson's correlation coefficient (CC) and the relative difference measurement star (*RDMS*). The *RDMS* is computed as

(6) $$\begin{array}{l} RDMS=\sqrt{{\sum_{k}\left(\frac{x_{k}}{\left\|x^{2}\right\|}-\frac{{\hat{x}}_{k}}{\left\|{\hat{x}}^{2}\right\|}\right)}^{2}} \end{array}$$

Two versions of both metrics can be used: (i) temporal version: for each node, the CC (or *RDMS*) is computed using all the time instants, and the mean and standard deviation of the CC (or *RDMS*) across nodes are then computed; and (ii) spatial version: for each time instant, the CC (or *RDMS*) is computed using all the nodes and the mean and standard deviation for the CC (or *RDMS*) across time instants are then computed. We tested both approaches and compared the results with the real and estimated epicardial maps, along with the error maps. We selected the temporal version since it showed more stability (less variance) and a more coherent behavior with the error maps.

#### 2.4.2. Dominant frequency metrics

The DF estimation is assessed by averaging the relative absolute error (*RAE*) in each node:

(7) $$\begin{array}{l} \text{RAE}\left(\%\right)=\frac{100}{N}\overset{N}{\underset{n=1}{\sum }}\frac{\left|F_{n}-{\hat{F}}_{n}\right|}{F_{n}} \end{array}$$

where *F*_*n*_ and ${\hat{F}}_{n}$ are the real and estimated DF for the *n*-th node, computed as described in Section 2.3.

#### 2.4.3. Phase and SP location metrics

The accuracy of the phase estimation is measured by using the CC and the *RDMS* between the phase maps. Regarding the SP location, the true SP position varies with time, but for the clinical practice it is useless to know its location at every time instant. Instead, it is more useful to estimate the probability of the SP being at each location (Haissaguerre et al., 2014). Therefore, we present a procedure to assess the location accuracy during an observation window. First, we estimate the SP location for each instant (see Section 2.3) and build a spatial histogram that represents the number of times the SP has been observed at each node. This histogram is normalized to obtain a spatial mass function (SMF) of the SP location, *p*(*n*), with *n*∈{1, …, *N*} the node index (see **Figure 11** for some examples). Second, defining the *SP region* as the region where the SMF is non-zero, we compare the real and estimated SMFs (*p*(*n*) and $\hat{p}\left(n\right)$, respectively) using four metrics: the weighted under-estimation indicator (WUI), defined as the percentage of the true SP region that is not detected out of the entire true SP region; the weighted over-estimation indicator (WOI), defined as the percentage of the misjudged SP region out of the estimated SP region; the correlation coefficient between SMFs (CC_*SMF*_), and the mode distance (MD) defined as the distance between the modes of the real and estimated SMFs. The first two metrics are weighted versions of those presented in Wang et al. (2013), which were used for ischemia region detection and were suitable for binary results (ischemia was present or not). Our estimate is probabilistic, so we weight the area *A*_*n*_ associated with the *n*-th node with the probability of locating the SP in that node, i.e., with *p*(*n*). The area associated with one node is the area of the faces surrounding the node, and it is needed since the triangulation of the epicardial surface is highly irregular, and then some faces are much bigger than others. With this weighting the performance metrics are computed as:

(8) $$\begin{array}{r} WUI\left(\%\right)=100\frac{\underset{n\in FN}{\sum }p\left(n\right)A_{n}}{\underset{n\in FN}{\sum }p\left(n\right)A_{n}+\underset{n\in TP}{\sum }p\left(n\right)A_{n}} \end{array}$$

(9) $$\begin{array}{r} WOI\left(\%\right)=100\frac{\underset{n\in FP}{\sum }\hat{p}\left(n\right)A_{n}}{\underset{n\in FP}{\sum }\hat{p}\left(n\right)A_{n}+\underset{n\in TP}{\sum }\hat{p}\left(n\right)A_{n}} \end{array}$$

where *FN* (False Negative) is the set of nodes belonging to the true SP region but not to the estimated region, *TP* (True Positive) is the set of nodes in both the true and estimated SP regions and *FP* (False Positive) the nodes belonging to the estimated SP region but not to the true SP region. The CC_*SMF*_ provides a direct comparison between both SMFs and aims at summarizing the WOI and WUI information in only one parameter. Finally, the MD is computed as the Dijkstra distance between the modes of the estimated and real SMFs. Since the SMFs are usually formed by unconnected areas (see **Figure 11** for some examples) the center of the SMF is not representative. However, the mode of the SMF is located in the area where the SP is placed with highest probability (red areas in **Figure 11**). This metric complements the WOI, WUI, and CC_*SMF*_ for those cases where the real and estimated SMFs are near but do not overlap.

### 2.5. Statistical method for comparisons

The performance metrics described in the previous section present different distributions that are not Gaussian. Then, for comparing results a Wilcoxon test is used (Hollander et al., 2013), which is a non-parametric statistical test that compares the median of the distributions of two samples. The null hypothesis is not rejected when no significant difference between the sample medians are found. Hence, in Section 3 the statistical significance of the difference between two results is assessed in terms of the Wilcoxon test at the 0.05 significance level, and the obtained *p*-value (*p*) is provided if necessary.

### 2.6. Experimental setup

Several *setups* have been considered for the experiments. Each *setup* is a combination of the following options:

- Algorithms: six Tikhonov algorithms: Tik-*mo*, where *m*∈{*g, i*} is the method for computing the regularization parameter (global or instantaneous) and *o* ∈ {1, 2, 3} represents the order of the regularization; three TSVD methods: TSVD-*o*, where *o* is the order of the regularization; DSVD (0-th order); GMRES; TV; Bayes and GS (with first order Tikhonov).
- SNR: four values (10, 20, 30, 40 dB).
- Models: three realistic models for cardiac activity (SR, SAF, and CAF).
- Targets: four parameters are estimated (epicardial potentials, DF, phase maps, and SP location).
- Performance metrics: for each target, different performance metrics are analyzed (epicardial potentials: CC, *RDMS*; phase: CC, *RDMS*; dominant frequency: *RAE*; SP location: SMF of SP location, WUI, WOI, CC_*SMF*_, and MD.

Since the number of possible *setups* is high, many results have been obtained. Due to space limitations, only the most relevant have been included in the Results section. Accordingly, unless otherwise stated, results have been obtained for SNR = 20 dB. Additional results can be found in the Supplementary Materials.

The free parameters for each algorithm were tuned as follows:

- Tikhonov and Tikhonov-based methods: the *l*-curve method was used for adjusting the regularization parameter α. For this purpose the regtool toolbox (Hansen, 1994) was used. This tool failed in providing a good value for α in many cases (especially with the SAF and CAF models). Then, the search space for the parameter was constrained to avoid extreme values.
- Bayes: in order to compute the a priori information, i.e., *C*_*x*_ in Equation (5), we randomly collected 150 samples, where a sample is the vector of the epicardial potentials at a specific time instant and all the nodes. These samples are collected from a 1-s window outside the window used for estimating the epicardial signal. Note that we are assuming a perfect knowledge of the epicardial potentials in all the nodes of the atria, which is an unrealistic spatial resolution for clinical recordings. This clearly biased the results obtained with the Bayes method and provided an upper bound on its performance.
- TSVD and DSVD: the number of singular values that are filtered out (TSVD) and the regularization parameter (DSVD) were computed by using the *l*-curve with the same constraints as those used for the Tikhonov-method.
- GMRES: 30 iterations were run, and the solution with minimal residuals was used.

## 3. Results

### 3.1. Epicardial potentials reconstruction

**Figure 5:**
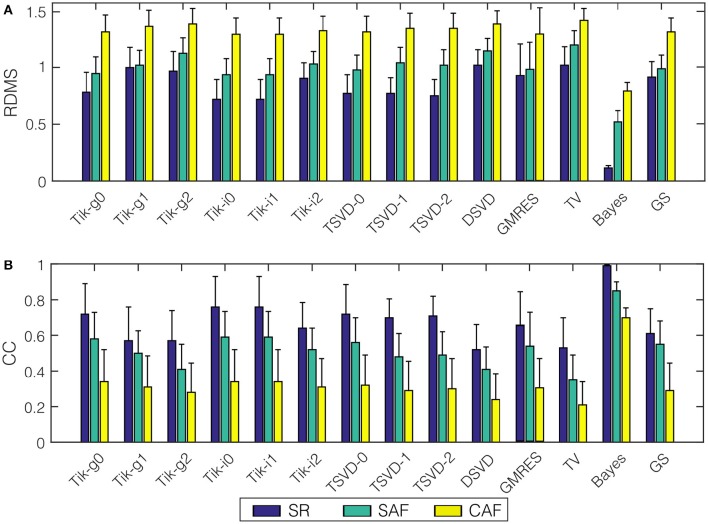
**Performance metrics for epicardial potentials reconstruction with all the algorithms and the three models: (A) ***RDMS***, (B) CC**. Vertical lines represent standard deviation. Models are represented with colors: SR (blue), SAF (green), CAF (yellow).

We run the 14 algorithms listed in the previous section to estimate the epicardial potentials for the three realistic models. Figure 5 shows the mean and standard deviation of the *RDMS* (Figure 5A) and CC (Figure 5B). Both metrics provided consistent information about the performance of each algorithm. Regarding the models, the best results were obtained for SR. The degradation of the metrics for the SAF model was lower than that for the CAF model (in terms of CC, between 0.06 and 0.22 for SAF and between 0.26 and 0.42 for CAF), and in all the cases it was statistically significant (*p*-values below 10^−6^ for SAF and even much smaller for CAF). The only exception is the Bayes method that, although degraded for the fibrillatory models, provided a very good performance for the three models (CC was 0.99, 0.85, and 0.70 for SR, SAF, and CAF, respectively). Recall that this method included a priori information taken from the epicardium. Among the rest of the algorithms, there was not a clear winner for all the models and metrics. The Tikhonov-based methods behaved similarly, although zero-order Tikhonov slightly outperformed the others. The instantaneous versions of the Tikhonov methods presented a slight gain with respect their global counterparts. For the fibrillatory models (SAF and CAF) this gain was always lower than 0.11 in terms of CC and 0.09 in terms of *RDMS*. For the best method (zero-order Tikhonov) the difference was not statistically significant nor in terms of CC (*p* = 0.10 and 0.73 for SAF and CAF, respectively) neither in terms of *RDMS* (*p* = 0.17 and 0.08 for SAF and CAF, respectively). This question is further analyzed in Section 3.4. The GS method also performed well for all models, but did not outperformed the Tik-i0 solution. Although not shown here, the results for the rest of SNR values were consistent with the ones in Figure 5, and can be found in the Supplementary Materials. Section 3.4 briefly discusses the impact of the SNR in the different metrics proposed in this work.

**Figure 6:**
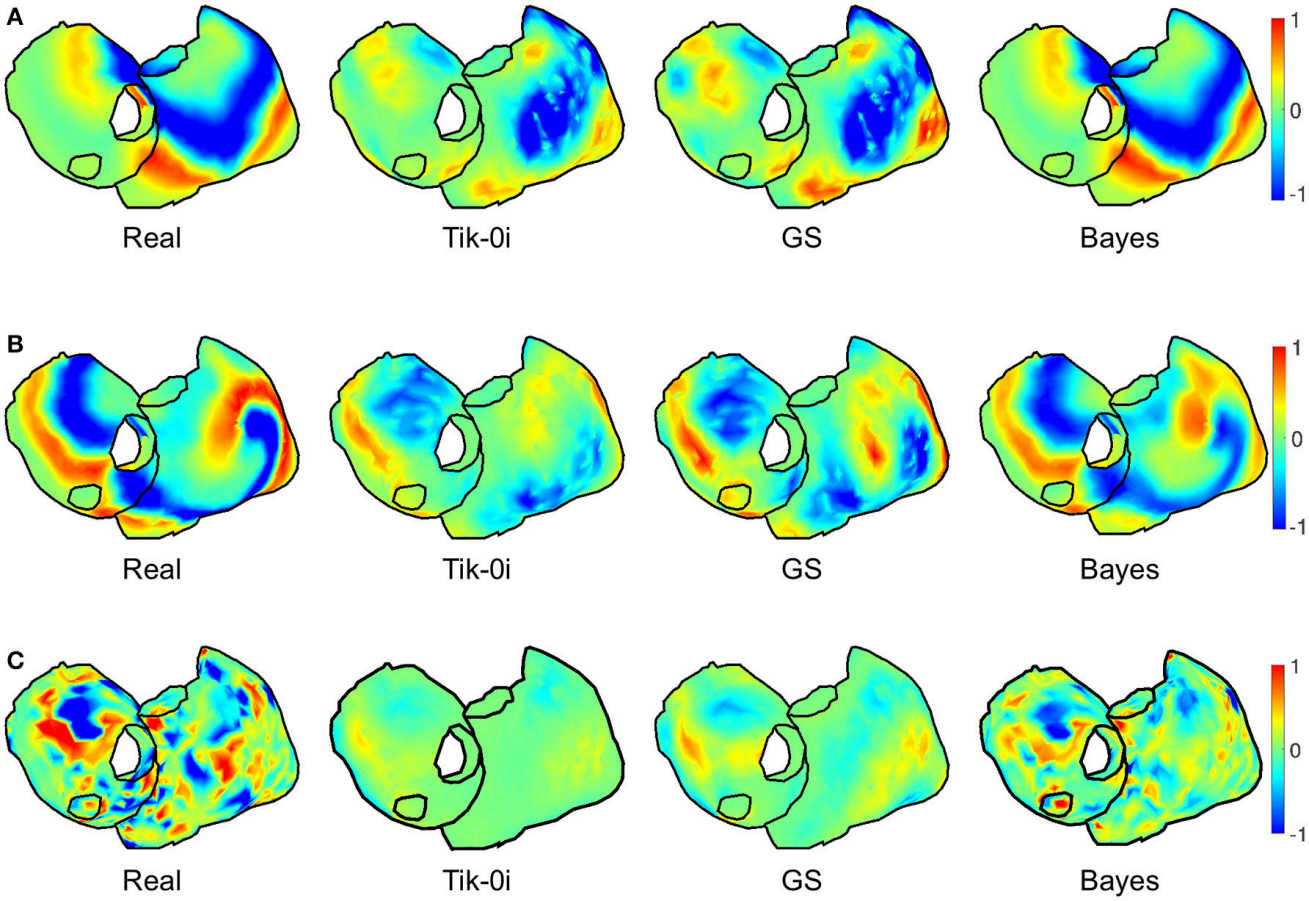
**Real and estimated potentials with Tik-i0, GS, and Bayes, for (A) SR, (B) SAF, and (C) CAF models**.

Figure 6 presents the actual and estimated maps of the epicardial potentials at one sample time instant. For the sake of clarity, in the following only a subset of three representative algorithms have been selected for figures: Tik-i0, (best known technique), GS (includes temporal regularization), and Bayes (includes a priori information). Moreover, only one view of both atria is represented. The panels in Figure 6 are consistent with the results in Figure 5. The Bayes algorithm was able to extract the basic pattern of the epicardial propagation, while Tik-i0 and GS blurred the epicardial maps for all the models. Regarding the activity complexity, the estimated maps for the CAF model almost completely faded the propagation patterns.

### 3.2. Dominant frequency

**Figure 7:**
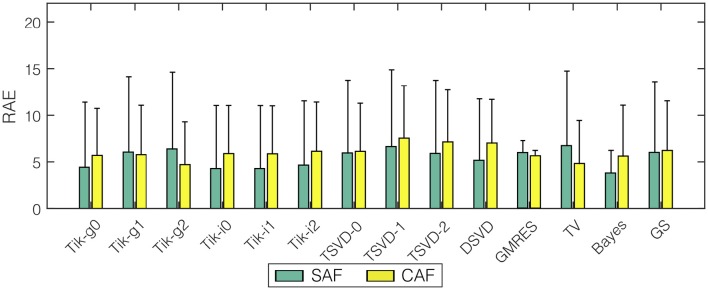
**Mean ***RAE*** for dominant frequency reconstruction with all the algorithms and the two fibrillatory models: SAF (green), CAF (yellow)**. Vertical lines represent standard deviation.

The DF in each node is computed from the estimated potentials using the method described in Section 2.3. Figure 7 shows the mean and standard deviation of the *RAE* for all the algorithms and the two fibrillatory models. The error for all methods was between 3.82% (Bayes, SAF) and 7.57% (TSVD-1, CAF), so even for the methods with poorest performance in the epicardial potentials reconstruction the reconstruction of DF was really accurate.The degradation for the CAF model with respect to SAF was also lower than that for the estimated potentials, although it was statistically significant for all the algorithms (*p*-values below 1.7·10^−3^). The variance of the error among nodes was high (note standard deviations above 10% in most cases). The best algorithm was Bayes, although the relative differences among algorithms were much lower than in the epicardial potential case.

**Figure 8:**
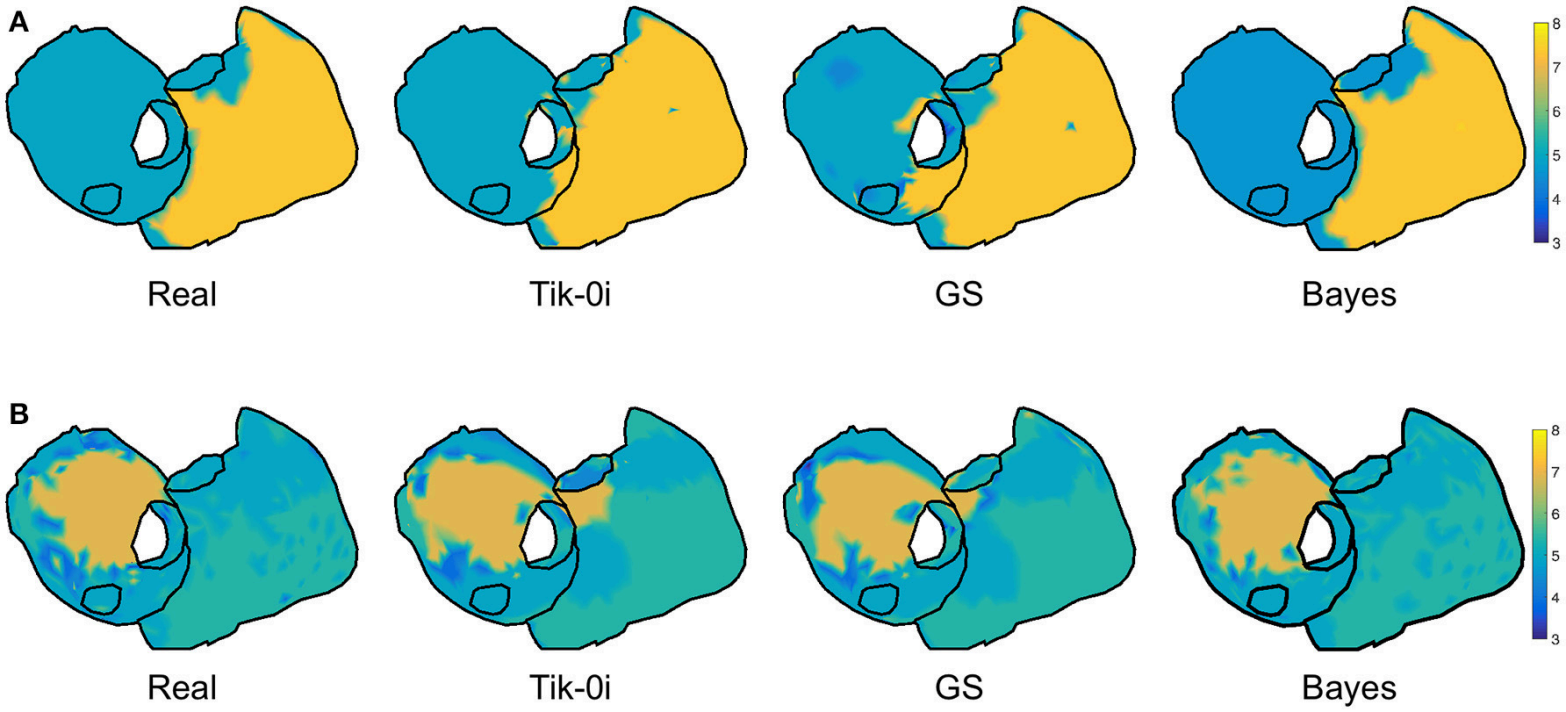
**Real and estimated DF with Tik-i0, GS, and Bayes, for (A) SAF and (B) CAF models**.

Figure 8 shows the maps for the DF at a sample time instant for the three representative algorithms and the two fibrillation models. The original spectral pattern consisted in two well-defined areas with high and low frequencies, respectively. For both models and all the methods, the estimated frequency maps are capable of capturing this pattern accurately. The comparison between this figure and Figure 6 highlights the fact that the DF estimation is more robust than the estimation of potentials, which becomes more evident when more complex fibrillation activity is present (like in the CAF model).

### 3.3. Phase maps and singularity point location

**Figure 9:**
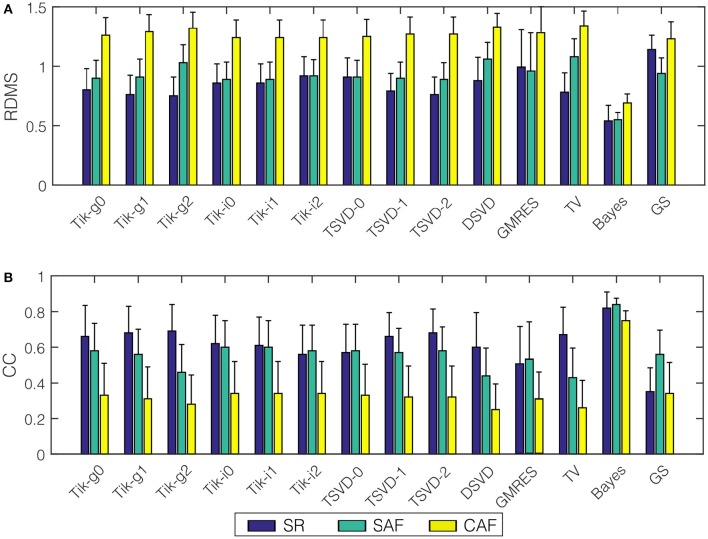
**Performance metrics for phase maps reconstruction with all the algorithms and the three models: (A) ***RDMS***, (B) CC**. Vertical lines represent standard deviation. Models are represented with colors: SR (blue), SAF (green), CAF (yellow).

**Figure 10:**
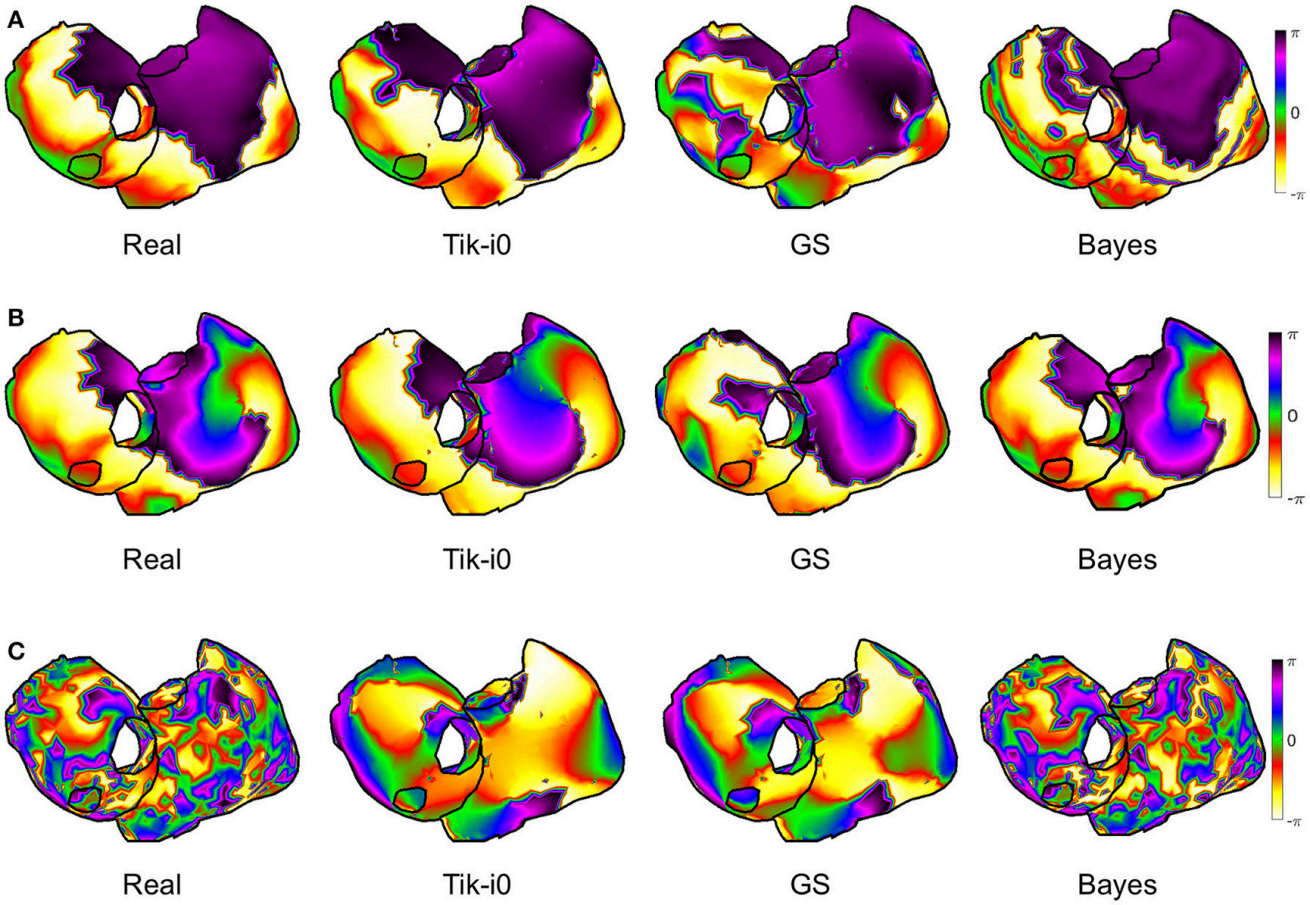
**Real and estimated phase maps with Tik-i0, GS, and Bayes, for (A) SR, (B) SAF, and (C) CAF models**.

In order to locate the activity drivers, the phase of the epicardial potentials must be computed. Figure 9 shows the mean *RDMS* (Figure 9A) and the CC (Figure 9B) for the phases computed from the estimated epicardial potentials, along with their standard deviation. As in the epicardial potentials case, the performance degraded for the fibrillatory models, especially for the CAF model: the maximum degradation in terms of CC was 0.24 and 0.41 for the SAF and CAF models, respectively, and the differences where statistically significant for all the algorithms except for Tik-i2 and TSVD-0 in the SAF case (*p* = 0.2 and 0.9 for CC, respectively, and *p* = 0.1 and 0.9 for *RDMS*, respectively). The Tikhonov algorithms, along with the TSVD ones and the DSVD provided similar performance, although the best algorithm was again Bayes. Figure 10 shows the phase maps for the three models and the three representative methods. As previously explained, in all the cases the Bayes method clearly outperformed the others. Performance degraded severely for the CAF model, except for the Bayes method. However, for both models and the three algorithms a singularity point was observed where the reentrant activity was placed.

**Figure 11:**
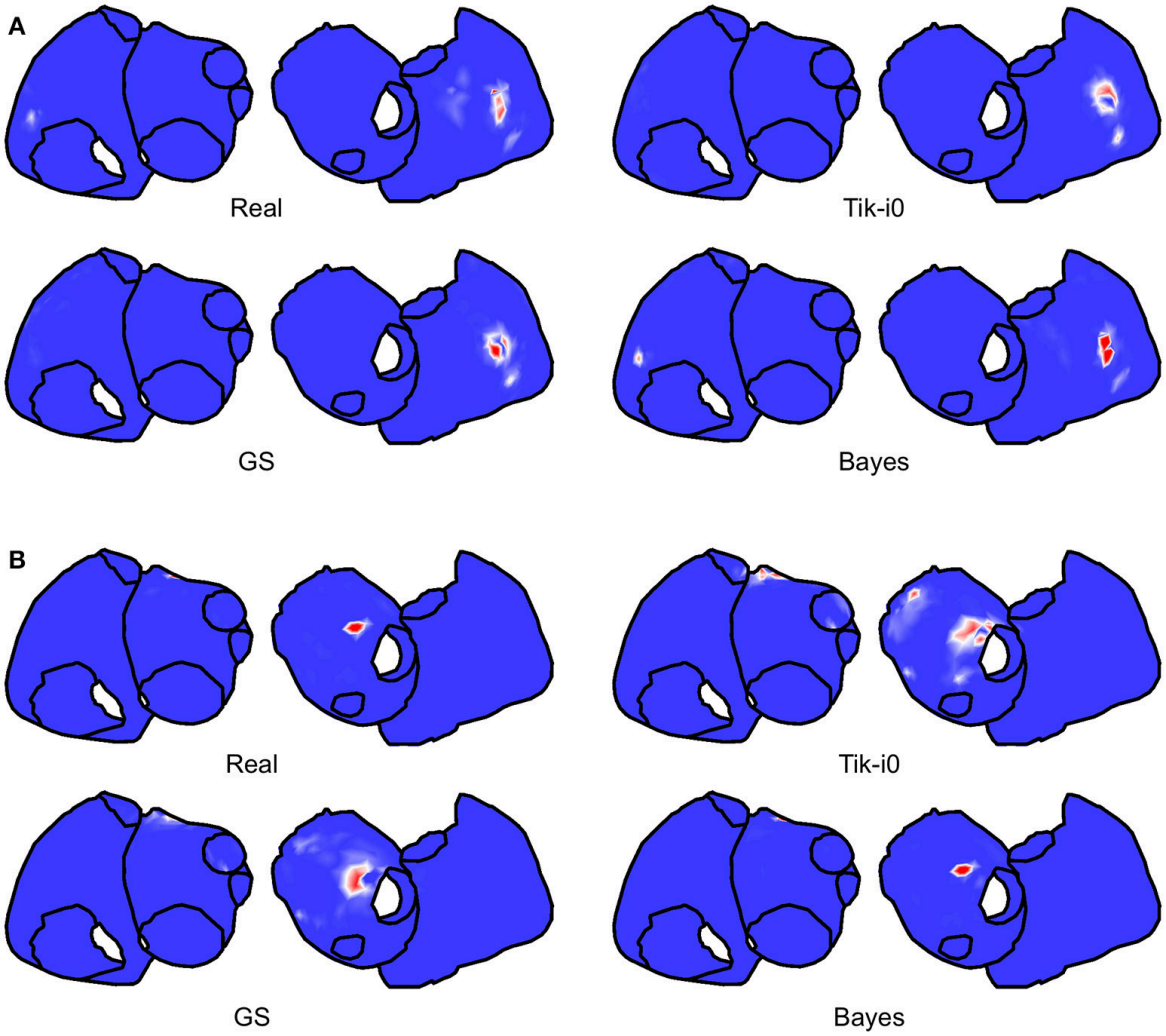
**Real and estimated SMFs of the SP location with Tik-i0, GS, and Bayes, for (A) SAF and (B) CAF models**.

Now we examine the results for the SP location. The three metrics described in Section 2.4 are computed for each method and model. Figure 11 shows the real and estimated SMF of the SP location. Since the estimated SP location could be in the posterior walls, both faces are represented in each panel. For the SAF model the SP was placed in the right atrium, and moved over a wide region (note the white areas for the upper right panel of Figure 11A). The three methods were are able to locate the SP in the right place most of the time (see the red areas in all the cases). For the CAF model the SP was placed in the left atrium. In this case the locations estimated with Tikhonov and GS methods were more spread out than in the Bayes case. However, even in these cases, with high probability the SP was located at the right place.

**Figure 12:**
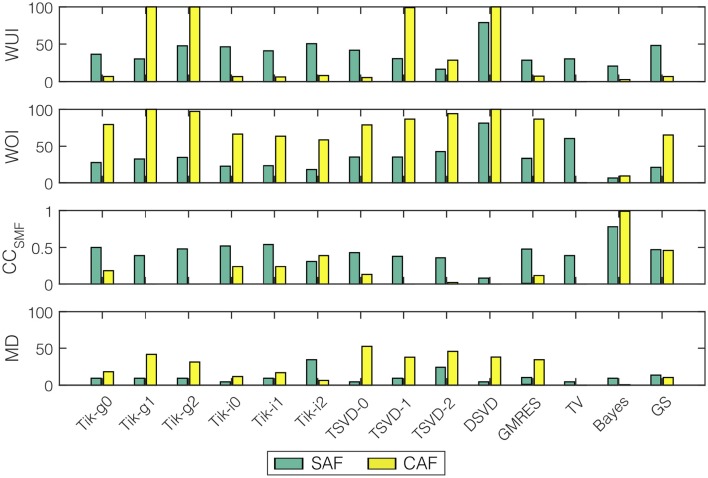
**Performance metrics for SP location and all the algorithms (WUI, WOI, CC_***SMF***_, and MD) for the two fibrillatory models: SAF (green), CAF (yellow)**. Cases where no SP was detected are marked with a cross.

To quantify the performance of these methods, the WUI, WOI, CC_*SMF*_, and MD metrics are shown in Figure 12. The cases when no SP was found inside the highest DF region are marked with a cross. Bayes and most Tikhonov-based methods (all but Tik-i2) performed well in terms of the four metrics for the SAF model (WUI between 20.68 and 47.65%, WOI between 6.53 and 34.39%, CC_*SMF*_ between 0.39 and 0.78, and MD between 4.52 and 9.33). The results varied importantly for the CAF model. While Bayes method still provided good results, Tikhonov methods overestimated the *SP region*. Note that the true *SP region* was small in the CAF case, so slight errors in the SP location led to high variations in the WOI, WUI and CC_*SMF*_ metrics. Also for this model, the MD increased respect to the SAF case. Finally, note that the effect of the two region-based metrics (WUI and WOI) is well summarized by the CC_*SMF*_, that is, CC_*SMF*_ tends to zero for high values of WUI and WOI. When the real and estimated SMFs did not overlap (e.g., DSVD for SAF and Tik-g1, Tik-g2, TSVD-1, DSVD for CAF) the WUI, WOI, and CC_*SMF*_ metrics provided a very bad result, but the MD quantified the distance between the modes of both SMFs.

### 3.4. Practical issues

#### 3.4.1. Regularization parameter

We have compared instantaneous and global versions of Tikhonov methods. To further analyze this issue, we benchmarked both zero-order Tikhonov methods (Tik-i0 and Tik-g0) with the regularization parameters obtained by using the L-curve and with their optimal values. These ones have been selected as the ones minimizing the *RDMS* within a fine logarithmic grid of 1000 points (each time instant for Tik-i0 and globally for Tik-g0). The experiment was run for the SAF model with SNR = 20.

**Figure 13:**
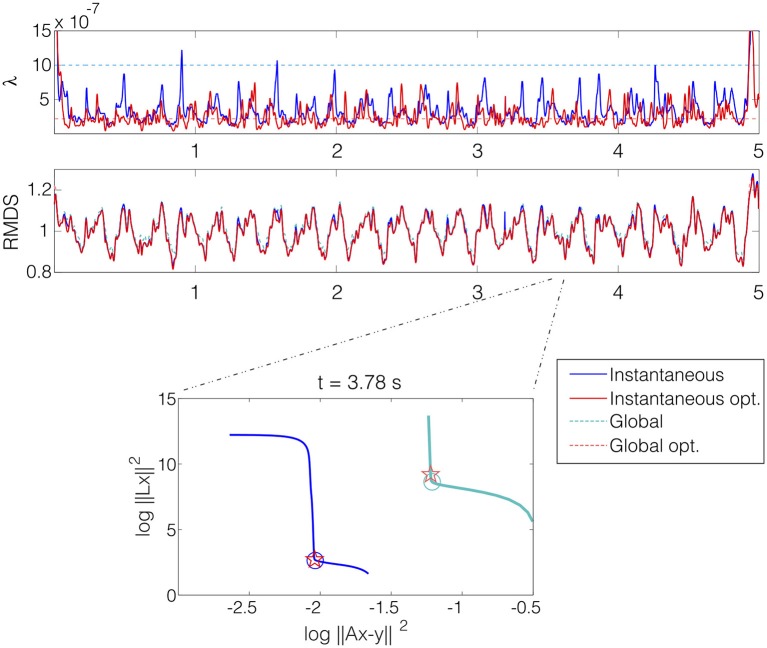
**The upper plot shows the regularization parameter λ selected for each time instant with four methods: instantaneous (blue), global (light blue), instantaneous optimum (red), and global optimum (light red)**. Middle plot shows the *RDMS* in time for that selection methods. Lower plot shows the *l*-curves for the four selection methods at time instant *t* = 3.78 s with the selected values marked with circles (L-curves) and stars (optimal). SAF model with SNR = 20.

Figure 13 shows the regularization parameter (upper plot) and the *RDMS* (middle plot) for each time instant. While the regularization parameter differed almost up to a decade for the four methods, the *RDMS* remained almost identical. The bottom panel shows the L-curves for the time instant with the highest difference between the instantaneous and global parameter (*t* = 3.78 s). For that time instant, despite this difference, the relative difference in terms of *RDMS* was only 6% between the Tik-i0 and Tik-g0, and the differences for the optimal values and those obtained with the L-curves were negligible. These results were consistent for both fibrillatory models and all SNRs.

#### 3.4.2. SNR

All the results in this section have been obtained for SNR = 20 dB. The whole set of results for SNR from 10 to 40 dB are presented in the Supplementary Materials. The behavior of the algorithms with respect to the SNR was consistent, i.e., there was a moderate degradation of the results for low SNRs for all the performance metrics, algorithms, and models. This degradation was higher for the metrics related to temporal targets than for the estimation of DF. For example, for the Tik-i0 algorithm, the relative degradation in terms of the CC of the epicardial potentials was 26 and 29% for the SAF and CAF models, respectively. However, the degradation in terms of the *RAE* of the dominant frequency was 9.8 and 11% for the same models. However, the SP location accuracy was sensitive to noise level, and degradation went up to 54 and 200% for both models in terms of the CC_*SMF*_. Finally, we observed that all the algorithms presented a similar response to variations of the SNR.

#### 3.4.3. Imperfect knowledge of the transfer matrix

It is important to note that the inverse methods used in this work have assumed a perfect knowledge of the transfer matrix *A*, which produces a bias in the results. If the estimation of *A* is not perfect a degradation of the results is expected. Although a deep analysis of this issue is out of the scope of this work, we have run an experiment for which we have added an amount of error to the transfer matrix in order to test the sensitivity of the Tik-i0 method. The error added is i.i.d Gaussian with a power computed to obtain a given SER (Signal to Error Ratio) in each row of the matrix. We tested two values of the SER (20 and 40 dB) with SNR = 20 and the SAF model, and compared the results with those obtained with no errors in the transfer matrix. The main results are shown in the Supplementary Materials and can be summarized as follows: as expected, the performance of Tik-i0 degraded as the error in the transfer matrix increases (CC = 0.40, *RDMS* = 1.08 and *RAE* = 9.34 for SER = 40, and CC = 0.19, *RDMS* = 1.27 and *RAE* = 15.68 for SER = 20). These results suggest that this issue should be further analyzed in future works.

#### 3.4.4. Temporal and spectral metrics

**Figure 14:**
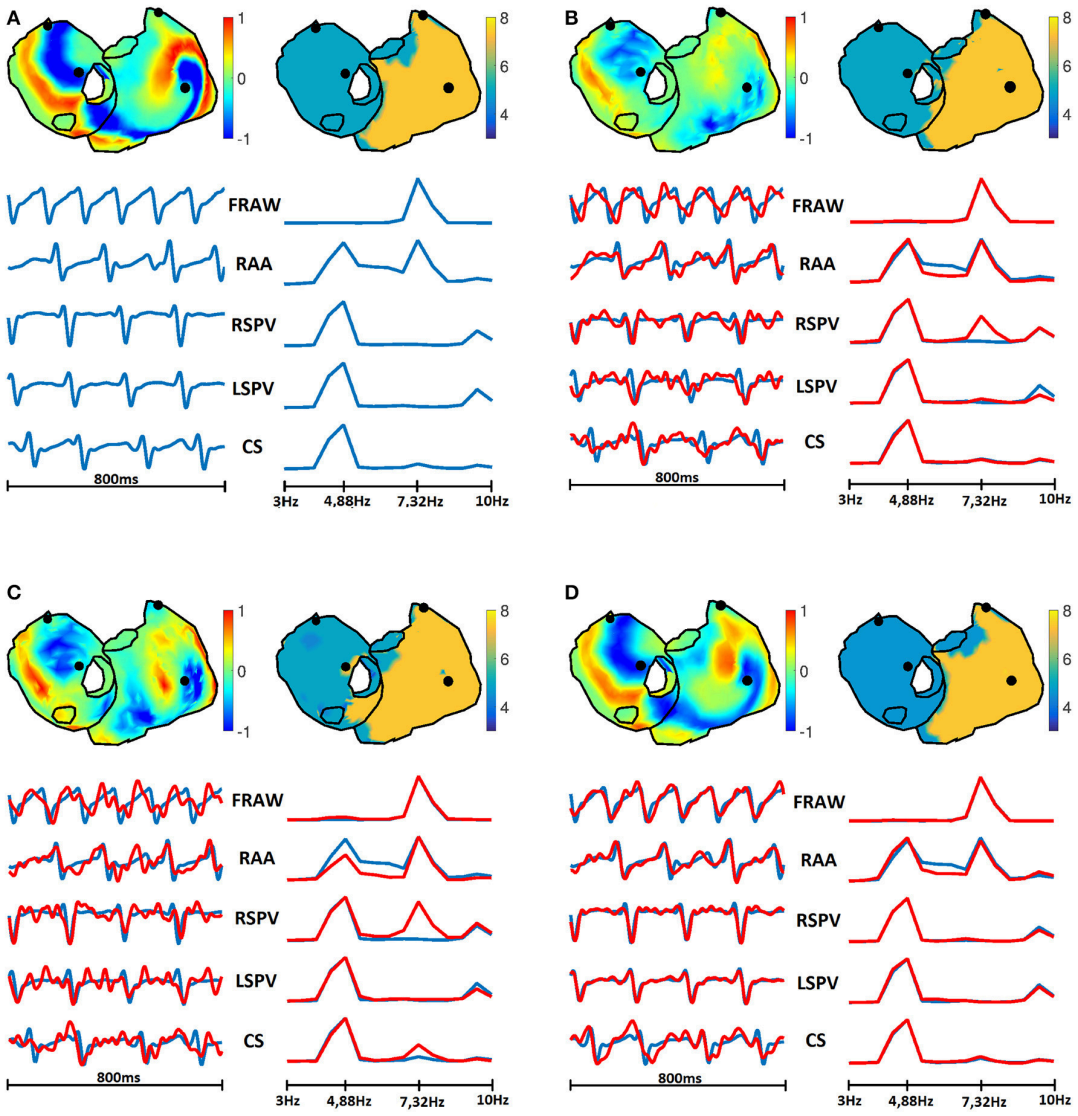
**Real (A) and estimated signals with Tik-i0 (B), GS (C), and Bayes (D) methods, for SAF model**. In each panel: potentials (top left) and DF (top right) maps; and temporal signals (bottom left) and their spectra (bottom right) for the five sample nodes presented in Section 2.1: RAA, CS, LSPV, RSPV, and FRAW. Real signals are in blue, while estimated are in red.

**Figure 15:**
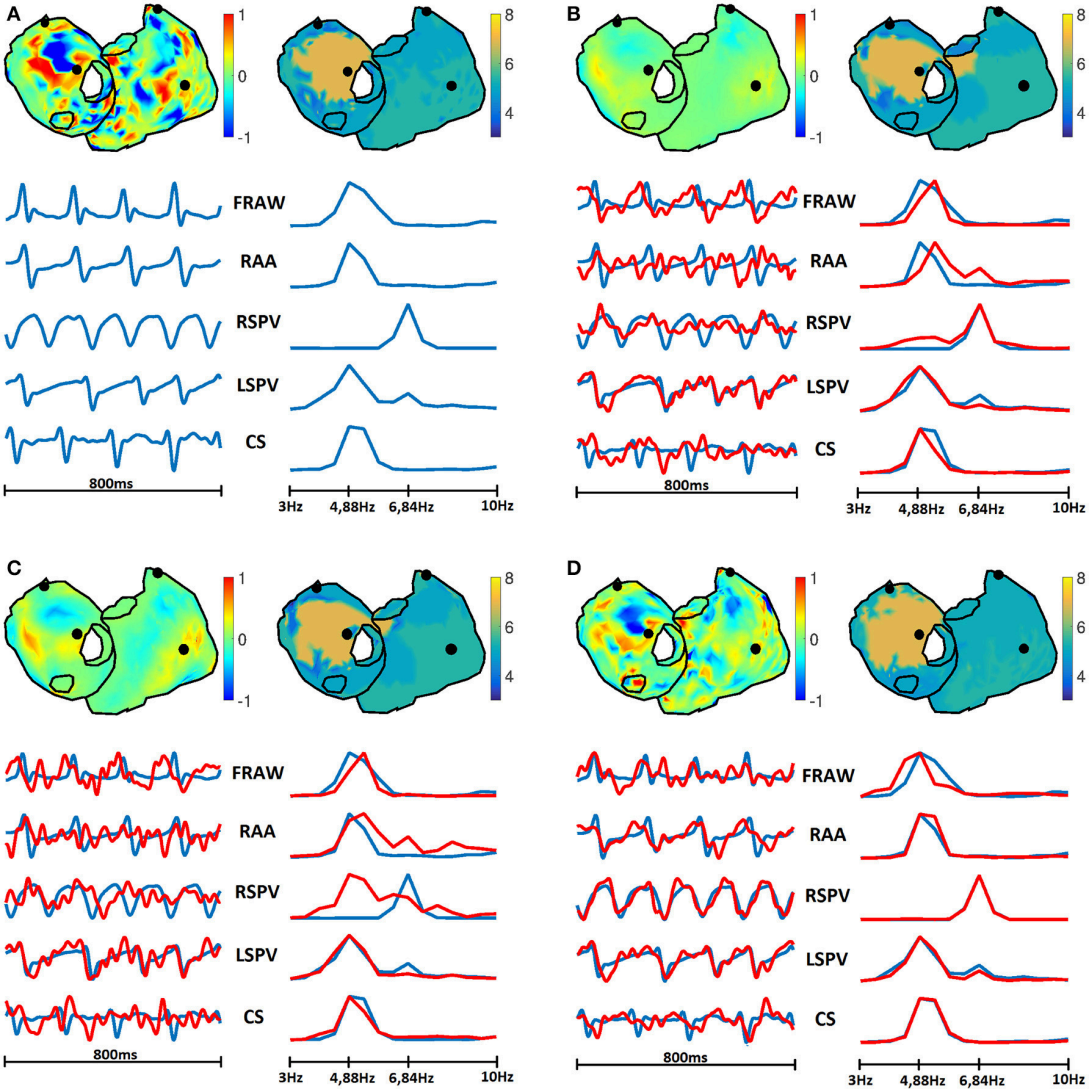
**Real (A) and estimated signals with Tik-i0 (B), GS (C), and Bayes (D) methods, for CAF model**. In each panel: potentials (top left) and DF (top right) maps; and temporal signals (bottom left) and their spectra (bottom right) for the five sample nodes presented in Section 2.1: RAA, CS, LSPV, RSPV, and FRAW. Real signals are in blue, while estimated are in red.

One of the purposes of this work was to identify a set of performance metrics that were able to characterize the utility of the different inverse methods in the clinical practice. Figures 14, 15 show a summary of the results obtained for the SAF and CAF models, respectively, with three representative algorithms and in the time and frequency domains. The potential and DF maps have been presented, along with time signals and their spectra for the five sample nodes described in Section 2.1. While there was a clear difference between the estimated and real time signals (see for example Tikhonov and GS methods for both models) the estimation of the DF was much more accurate. Only the estimated spectra of the signals recorded at the RSPV differed from the real ones, since this point is placed near the boundary that separates the regions with different DFs.

## 4. Discussion

In this work we have assessed a plethora of methods for the inverse problem of electrocardiography in AF conditions. Specifically, 14 techniques have been used to estimate different clinical targets for three computational models, each one with a more complex electrical activity. Results have been obtained for different SNRs, and a complete set of performance metrics have been computed and benchmarked. Practical considerations have also been explored.

In fibrillatory conditions most of the algorithms performed similarly when estimating the epicardial potentials. The difference in terms of *RAE* was even lower (at most 7 percentual points between the worse and best methods), since all the methods performed quite well when estimating the dominant frequency. These results suggest that for complex propagation patterns simpler algorithms may provide a similar error than that obtained with more sophisticated techniques. Results also showed that if a priori information about the second order statistics of the epicardial potentials was available, the Bayes method presented a clear advantage in all the cases, which motivates a further search of methods that uses any a priori additional information about the electrical activity in the patient heart in AF conditions.

Regarding the clinical targets, maps in Figures 6, 8, 11 show that the estimation of dominant frequency and SP location are more robust than the estimation of epicardial potentials, despite the fact that the former are computed from the latter. When more complex propagation patterns were present, the epicardial potential estimation degraded severely for all the methods. Even when the error of estimated potential was high, the spectral information was still accurately obtained. Since dominant frequency maps and SP location are more useful in the clinical practice than raw epicardial potentials, this is a promising result supporting those obtained in Haissaguerre et al. (2013); Pedrón-Torrecilla et al. (2016).

Several metrics have been computed for the different clinical targets. In the case of epicardial potentials, the CC and *RDMS* provided consistent information. *RDMS* presented less variance while CC is bounded between 0 and 1, so it is easier to interpret. In the case of dominant frequency estimation, although the used performance metric had high variance, it is simple to interpret and consistent with the information provided by dominant frequency maps. Finally, we proposed a methodology for benchmarking different techniques for SP location. The SMF of the SP location aggregates the results of the location procedure in a time-scale of a few seconds, and provides a simple representation of the location of the reentrant activity, which is very easy to interpret in a practical clinical environment. WUI, WOI, and CC_*SMF*_ metrics, along with the MD are also very representative and enables a quantitative comparison between real and estimated SMFs, so they provide an objective assessment of the SP location accuracy.

Some practical issues have been explored in this work. The comparison between global and instantaneous Tikhonov methods (with the optimal regularization parameters and those obtained with the L-curve) suggests that the Tikhonov (zero-order) method in AF is somewhat insensitive to moderate changes of the regularization parameter. Then, since the computation time of the L-curve is high, the use of the globally estimated parameter might be a useful alternative. Results for different SNRs showed that no algorithm was significantly more robust than others with respect to changes in noise level. Also, it turns out that dominant frequency estimation was more insensitive to SNR degradation. Finally, introducing errors in the transfer matrix notably degraded the performance of the reconstruction, and hence this topic should be further analyzed.

The experiments performed in this work relied on the accuracy of the computational models of the electrical activity in the atria. Despite the fact that our mathematical models continue presenting some differences with real data, the distribution of activation patterns represented by the real epicardial potentials maps follow the data observed by electrical intracardiac maps and experimentally by using optical mapping (Berenfeld et al., 2002).

## 5. Conclusions

As a summary, some important points may be concluded from the previous discussion. First, simple inverse methods may perform as well as more sophisticated versions when applied to complex fibrillatory patterns. Also, in our scenario the computation of instantaneous regularization parameter took a long time and is not critical in terms of performance, so it may be avoided. If a priori information is available, its usage (by means of Bayes framework or other technique) may provide a clear improvement in performance. Second, clinically useful targets (DF and SP location) can be accurately estimated even when epicardial potential estimation has poor quality, so further methods aiming to obtain spectral targets without the need of previously computing the epicardial potentials should be explored. Third, a complete set of performance metrics have been presented in order to assess inverse methods. Hence, the results obtained in this work suggest that when a priori information is available a Bayesian method incorporating it could be the best approach, and when it is not, a simple method like zero-order Tikhonov, even with a constant regularization parameter, is a good choice for solving the inverse problem in AF. By making public all the routines used in this work, we aim at facilitating the task of using a unified framework for inverse methods benchmarking.

## Author contributions

Experimental setup, code implementation, computational tests: CF, IH, MR, ÓB, FAA. Data collecting, cleaning, and pre-processing: VS, IH, AL, FA, MG, AC. Manuscript preparation: CF, VS, IH, MR, AL, FA, MG, AC, FAA.

### Conflict of interest statement

The authors declare that the research was conducted in the absence of any commercial or financial relationships that could be construed as a potential conflict of interest.
