# Corrigendum: Regularization Techniques for ECG Imaging during Atrial Fibrillation: A Computational Study

**DOI:** 10.3389/fphys.2016.00556

**Published:** 2016-11-17

**Authors:** Carlos Figuera, Víctor Suárez-Gutiérrez, Ismael Hernández-Romero, Miguel Rodrigo, Alejandro Liberos, Felipe Atienza, María S. Guillem, Óscar Barquero-Pérez, Andreu M. Climent, Felipe Alonso-Atienza

**Affiliations:** ^1^Department of Telecommunication Engineering, Universidad Rey Juan CarlosFuenlabrada, Spain; ^2^ITACA, Universitat Politécnica de ValenciaValencia, Spain; ^3^Instituto de Investigación Sanitaria Gregorio Marañón, Hospital General Univesitario Gregorio Marañón, Universidad Complutense-Facultad de MedicinaMadrid, Spain

**Keywords:** atrial fibrillation, ECG imaging, regularization, dominant frequency, rotor location, inverse problem

Due to an oversight, there was an error in the Authors' Contributions statement as published. Author “Víctor Suárez-Gutiérrez” has also contributed towards Experimental setup, code implementation, computational tests, this information went missing in the original article. The correct statement should read as mentioned below. The authors apologize for this mistake. This error does not affect the scientific conclusions of this article in any way.

## Author contributions

Experimental setup, code implementation, computational tests: CF, VS, IH, MR, ÓB, and FAA. Data collecting, cleaning, and pre-processing: VS, IH, AL, FA, MG, and AC. Manuscript preparation: CF, VS, IH, MR, AL, FA, MG, AC, and FAA.

### Conflict of interest statement

The authors declare that the research was conducted in the absence of any commercial or financial relationships that could be construed as a potential conflict of interest.

